# Recent Trends in Lateral Flow Immunoassays with Optical Nanoparticles

**DOI:** 10.3390/ijms24119600

**Published:** 2023-05-31

**Authors:** Jaehi Kim, Min-Sup Shin, Jonghyun Shin, Hyung-Mo Kim, Xuan-Hung Pham, Seung-min Park, Dong-Eun Kim, Young Jun Kim, Bong-Hyun Jun

**Affiliations:** 1 Department of Bioscience and Biotechnology, Konkuk University, Seoul 05029, Republic of Korea; 2 Molecular Imaging Program at Stanford (MIPS), Department of Radiology, Stanford University School of Medicine, Stanford, CA 94305, USA; 3 Department of Urology, Stanford University School of Medicine, Stanford, CA 94305, USA

**Keywords:** lateral flow immunoassay, diagnosis, nanoparticle, optical nanoparticles

## Abstract

Rapid, accurate, and convenient diagnosis is essential for effective disease management. Various detection methods, such as enzyme-linked immunosorbent assay, have been extensively used, with lateral flow immunoassay (LFIA) recently emerging as a major diagnostic tool. Nanoparticles (NPs) with characteristic optical properties are used as probes for LFIA, and researchers have presented various types of optical NPs with modified optical properties. Herein, we review the literature on LFIA with optical NPs for the detection of specific targets in the context of diagnostics.

## 1. Introduction

Human health is under constant threat by various factors, most notably diseases [1,2]. Mild diseases cause minor discomfort, whereas serious conditions, such as cancer, often lead to death. In light of the coronavirus disease 2019 (COVID-19) pandemic, the threat imposed by viral diseases considerably increased following late 2019 [3,4,5]. Various effective therapeutics have been developed in a bid to prevent or cure disease. However, not all treatments are curative, highlighting the importance of early diagnosis for effective disease management and patient survival. This has given rise to a plethora of diagnostic approaches [6].

Diagnostics are usually based on the detection of a causative pathogen or biomarker [7,8]. Enzyme-linked immunosorbent assay (ELISA) is frequently used for biomarker detection [9,10]. It has several advantages, such as quantification and its low limit of detection (LOD), high selectivity, accuracy, and reproducibility. Despite these merits, ELISA-based diagnostics are often limited by a long reaction time (4–6 h) and the need for specialized laboratory equipment and personnel [11]. Recently, lateral flow immunoassay (LFIA) has gained attention as an alternative to ELISA because of its low cost, short reaction time, and ease of use [12,13].

Probe selection is essential for the efficient detection or quantification of target biomarkers using LFIA [14]. In the early stages of LFIA development, organic dyes, such as fluorescein isothiocyanate, were used as probes. These dyes generate strong signals; however, they are also associated with photobleaching [15,16]. Researchers have, therefore, sought alternative probe materials, identifying nanomaterials as adequate candidates. As a result, the efficiency and convenience of LFIA diagnosis have been enhanced using various optical metal nanoparticles (NPs), such as gold NPs (Au NPs), carbon NPs, quantum dots (QDs), and upconversion NPs (UCNPs). This review discusses the recent trends in LFIA using optical NPs as probes for biomarker detection.

## 2. LFIA Assays with Optical NPs

### 2.1. Principle of LFIA

**Figure 1:**
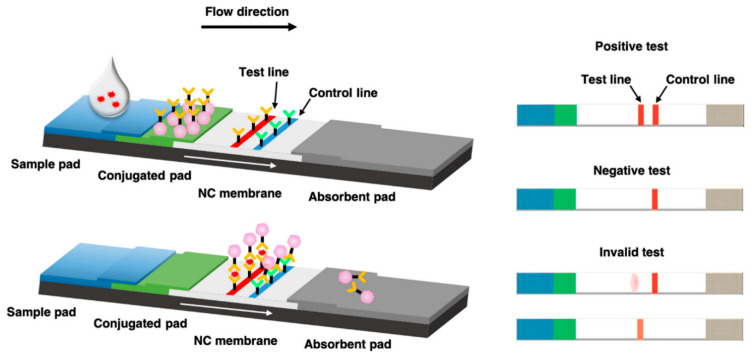
Schematic illustration of a lateral flow immunoassay (LFIA) kit for antigen detection [19].

The LFIA kit consists of sample, conjugate, and absorbent pads attached to a nitrocellulose membrane backbone (Figure 1) [17,18,19,20]. A liquid sample to be diagnosed is loaded onto the sample pad and developed toward the absorbent pad. When the developed sample reaches the conjugate pad, it is mixed with preloaded probes. Subsequently, samples and probes are developed together. Targets in the sample are bound and complexed with the probes by antibodies. Upon reaching the test line where target-capturing antibodies are immobilized, the target–probe complexes are captured and cannot be developed further. Other components in the samples and non-complex probes pass the test line, with non-complex probes captured at the control line by antibodies immobilized on the control line. The captured probes exhibit their characteristic optical signal at the corresponding lines, with the type of optical signal and signal intensities varying based on the probe type and optical properties. Thus, we reviewed papers on LFIA after classifying them based on the type of optical NPs used as probes in LFIA.

### 2.2. LFIAs with Au NPs

Owing to their excellent optical property, Au NPs are the most widely used optical nanomaterials in LFIA. The optical characteristic of Au NPs is derived from their localized surface plasmon resonance (LSPR), a physical phenomenon of collective oscillation of the electrons on Au NPs after being exposed to an incident light [21]. Au NPs are fabricated via a seed-mediated growth method, wherein Au seeds act as the core of Au NPs. Au seeds are usually fabricated using Turkevich’s method (approximately 15 nm) or Martin’s method (3 to 5 nm) [22,23]. Unlike the bulk form, Au NPs have unique optical properties and have been applied in bioimaging and biosensing [24,25]. Au NPs can be used as substrates for surface-enhanced Raman scattering, emitting the characteristic Raman signal of the target compound. In addition, well-dispersed Au NPs of sizes of 15 to 150 nm exhibited a red color in solution based on the LSPR effect. The color of Au NPs can be controlled by adjusting their size via their growth.

Khlebtsov et al. quantified and revealed the relationship between Au NP size and LOD [26]. They prepared spherical and monodisperse Au NPs of various sizes (from 16 to 115 nm) and characterized them. The Au NPs were dropped onto membranes with serial dilution and the signal intensity of each spot was measured. The LOD for each Au NP was inversely proportional to the 3.1 power of particle size per spot area.

One limitation of Au NPs is that the weak signal causes inadequate sensitivity. For this reason, efforts for signal enhancement have been reported. For example, accumulating more Au NPs at the test line is the simplest approach for signal enhancement. Shen et al. utilized polyamidoamine (PAMAM) dendrimers to induce the aggregation of Au NPs [27]. PAMAM, highly branched, tree-like, water-soluble, and multifunctional macromolecules, are attached to the Au NP surface via electrostatic interactions. Therefore, the authors dispensed and ran the conjugates consisting of PAMAM, antibodies, and multiple Au NPs, including multiple Au NPs. Their concepts were based on the fact that the signal produced by an aggregate of smaller Au NPs can be stronger than that produced by a larger individual Au NP. The assay results show that this approach has an LOD of 0.1 ng/mL and is 20-fold more sensitive than the conventional LFIA method using individual Au NP under identical conditions.

However, the Au NP assemblages are usually hard to precisely control in terms of number and final size, and Au NPs themselves tend to be naturally aggregated according to the environment. Therefore, other research groups suggested a method that sequentially ran two types of Au NP conjugates. Shen et al. described a dual Au NP conjugate-based LFIA using oligonucleotides [28]. The first Au NP (30 nm) was modified with DNA1 and the second Au NP (16 nm) was modified with DNA2 and aptamer. Therefore, they were bound specifically via hybridization between DNA1 and DNA2. This system was verified using thrombin as a potential biomarker, and the LOD was 0.25 nM, with a linear range of 0.5 to 120 nM. Similarly, Shen et al. developed the signal amplification method using dual gold Au NPs [29]. They separately prepared two Au NP conjugate types. The first were Au NPs modified with biotin and antibody and the second were Au NPs modified with streptavidin. They conducted the assay in a one-step process using two conjugation pads. They proved a concept to detect hepatitis B surface antigen (HBsAg), a biomarker of hepatitis B virus infection. The LOD of the system was 0.06 ng/mL, with a 30-fold enhancement compared to the conventional LFIA method.

Although the post-assay growth of the Au NPs was once tested as a signal enhancement method, its additional procedure for loading gold enhancers was laborious and time-consuming [30,31]. Panraksa et al. reported a one-step Au NP enhancement via sequential flow LFIA [32]. The authors designed the patterned nitrocellulose membrane consisting of two channels. Thus, all immunoreagents and gold enhancer solutions were delivered sequentially. With the gold enhancement system, the LOD for C-reactive protein (CRP) visualized by the naked eye was 0.1 μg/mL and the calculated LOD was found to be 0.001 μg/mL, with a linear range of 0.1 to 5 μg/mL.

**Figure 2:**
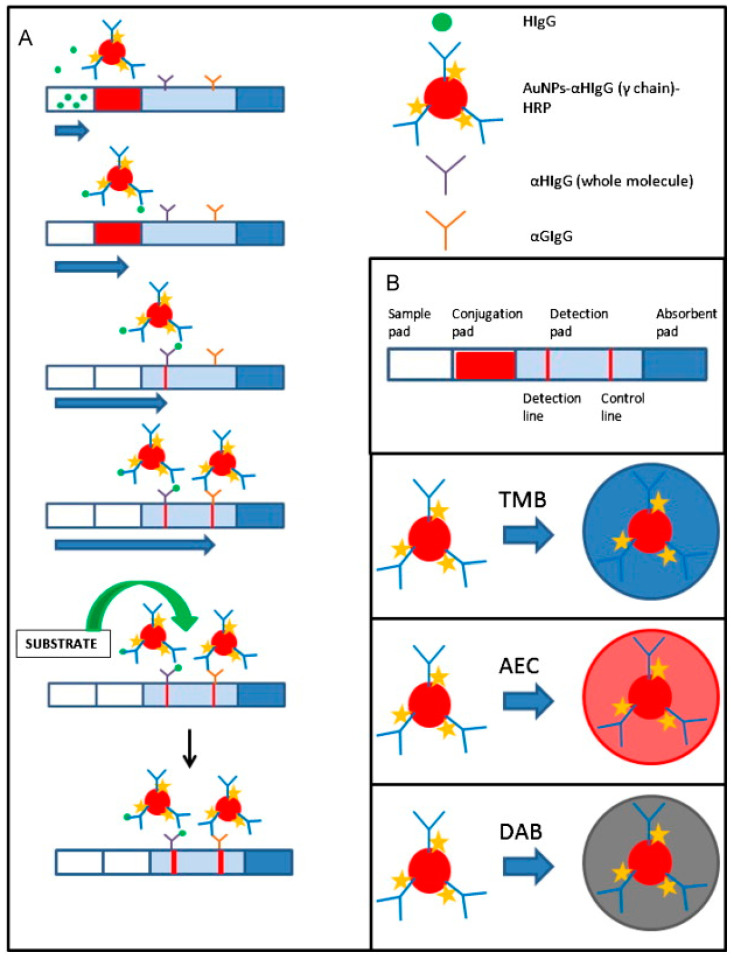
Schematic of the LFIA for human IgG detection. (**A**) Procedure of the assay. (**B**) Different parts of an LFIA strip and cartoons representing the gold nanoparticles (AuNPs) modified with an anti-human IgG γ chain-specific horseradish peroxidase (HRP)-modified antibody, with different colors expected for the different substrates (3,3′,5,5′-tetramethylbenzidine [TMB], 3-amino-9-ethylcarbazole [AEC], and 3,3′-diaminobenzidine-tetrahydrochloride [DAB]) used [17].

Meanwhile, there also was an enzymatic amplification inspired by traditional bioassays. Parolo et al. proposed a method for enhancing sensitivity by introducing horseradish peroxidase (HRP) onto the Au NPs (Figure 2) [17]. HRP was introduced onto Au NPs after conjugation to an antibody, whereafter the Au NPs were developed. After development, the fabricated particles were read, originating from the SPR effect, similarly to the Au NPs without HRP. The developed LFIA strips were dipped into three different HRP substrate solutions (3,3′,5,5′-tetramethylbenzidine [TMB], 3-amino-9-ethylcarbazole [AEC], and 3,3′-diaminobenzidine-tetrahydrochloride [DAB]), and the signal intensity of each LFIA strip was compared. The strips dipped in the TMB and AEC solutions showed a deeper color at the test line and this difference could be confirmed with the naked eye. In the case of the TMB-treated strip, the LOD was approximately 10-fold lower than that of Au NPs without HRP.

**Figure 3:**
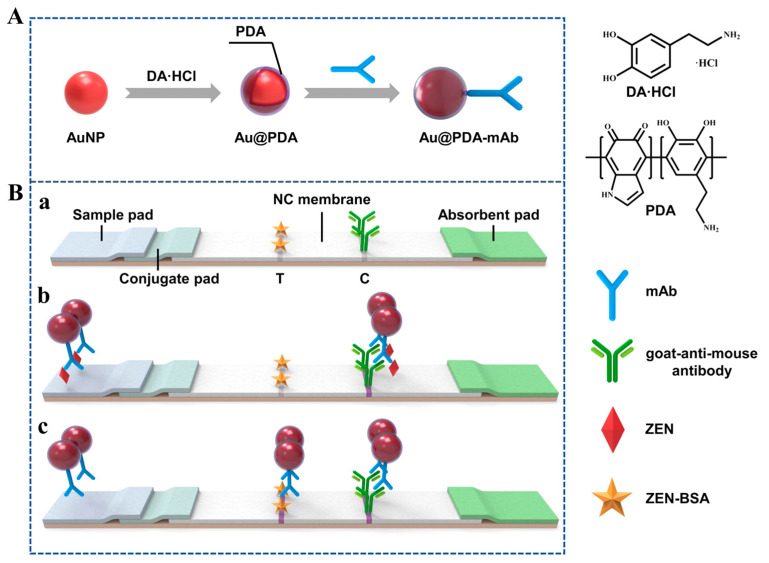
Principle of Au@PDA–LFIA. (**A**) Preparation of Au@PDA–mAbs. (**B**) Schematic illustration of ZEN detection using Au@PDA–LFIA: (**a**) composition of the test strip, (**b**) detection mode in the presence of ZEN, and (**c**) detection mode in the absence of ZEN. In this figure, T and C indicate test line and control line, respectively [33].

Another approach for enhancing sensitivity by coating Au NPs with polydopamine was reported by Xu et al. [33] (Figure 3). Polydopamine can easily adhere to the surface of Au NPs, with encapsulation enhancing their biostability and biocompatibility. Furthermore, polydopamine-coated Au NPs (Au@PDA-10) exhibited a deep purple color, which was stable even under basic or high-salt conditions, with the red color of uncoated Au NPs being fainter. Based on this optical property, the LFIA LOD for zearalenone, based on Au@PDA-10, was 10-fold lower than that based on traditional Au NPs (7.4 pg/mL and 76.1 pg/mL, respectively).

The effect of antibody introduction on Au NPs on the LOD was also studied previously. Di Nardo et al. [34] fabricated Au NPs and introduced an anti-cortisol antibody onto the surface of Au NPs in three ways: adsorption, covalent conjugation, and protein–protein interactions. Each Au NP was used for detecting cortisol using LFIA. The result revealed that the LOD was unrelated to the method of antibody introduction but depended only on the number of introduced antibodies.

### 2.3. LFIAs with Carbon NPs

Carbon NPs are comparatively inexpensive probes that can be easily prepared [35]. Since carbon NPs display high contrast against the background, they are advantageous in achieving highly sensitive tests without an additional enhancement strategy [36]. Linares et al. conducted a systematic study comparing the performance of Au NPs, latex particles, and carbon NPs in LFIA under identical conditions. The results showed that carbon NPs had a lower LOD than other NPs when other conditions were the same [37]. Another study also reported that the LOD of carbon NP-based LFIA was 12.5 times lower than that of Au NPs [38]. However, their hydrophobic nature often makes their bio-applications difficult [36,39]. The reduced colloidal stability, which causes irregularly shaped large particles, is an unfavorable characteristic in LFIAs [35]. The non-specific adsorption of the biomolecules on carbon NPs is hard to control; thus, their bioconjugation process is also labored compared to Au NPs [39].

Wiriyachaiporn et al. reported the carbon NP-based LFIA for detecting the influenza A virus [40]. The nanoprobes consisting of carbon NPs and monoclonal anti-influenza A nucleoprotein antibodies were loaded on the conjugate pads. The monoclonal anti-nucleoprotein and secondary goat anti-mouse IgG antibodies were spotted on the test line and control line, respectively. In optimal conditions, the system successfully detected the influenza A virus in influenza A-inoculated allantoic fluid and the LOD of the assay was 3.5 × 10^2^ TCID_50_/mL. More importantly, no cross-reactivity was identified in the selectivity test with various proteins and other viral proteins (influenza B and canine distemper virus).

Wang et al. proposed another carbon NP-based LFIA for detecting *Salmonella enteritidis* [41]. The authors introduced a novel sandwich strategy that requires only one kind of antibody (capture antibody) by replacing the role of detection antibody with electrostatic adsorption between the positively charged, nitrogen-rich carbon NPs and the negatively charged bacteria. The LOD of the assay was 10^2^ cfu/mL, with a linear range from 10^2^ to 10^8^ cfu/mL. Notably, the proposed format for sandwich LFIA showed comparable or better results than traditional sandwich LFIA.

The carbon NPs have been utilized for the development of LFIA during the COVID-19 pandemic. Ju et al. developed a novel fluorescent LFIA using aggregation-induced emission carbon dots [42]. The LFIA strip consisted of two control lines for IgM and IgG detection and one control line. The spike protein-attached carbon NPs were loaded on the sample pad. The mouse anti-human IgM, mouse anti-human IgG, and goat anti-IgY antibodies were sprayed on the two test lines and one control line. The prepared aggregation-induced emission carbon dots had dual emission properties (blue and red fluorescence). The LOD of the assay was 100 μg/mL and 100 pg/mL for IgM and IgG, respectively.

### 2.4. LFIAs with QDs

QDs are a type of semiconductor NPs that can emit fluorescence signals under ultraviolet light (UV) irradiation with a high quantum yield [43,44]. Unlike the bulk semiconductor, their electrons and holes, created by the absorption of photons, are confined because the diameter of QDs is smaller than its exciton Bohr radius. It is called the “quantum confinement effect”, wherein energy levels are quantized [45,46]. Based on quantum confinement effects, the wavelength of the emitted light is determined by the size of the QDs. The bandwidth of the emitted light spectra is narrower than that of fluorescent organic dyes, such as fluorescence I or rhodamine B [15]. Their excellence in terms of the brightness and stability offers an opportunity for sensitive and quantitative detection. Hence, they are considered a very promising probe in biosensing research. Furthermore, the color of emitted light can be varied by adjusting the size of the particles, providing the possibility of multiplex analysis with QDs [47,48].

Considering the above-described advantages, many research groups have developed LFIA systems using QDs. Bock et al. fabricated silica-coated CdSe@ZnS QDs via reverse microemulsion detecting prostate-specific antigen (PSA) via LFIA [49]. An anti-PSA antibody was then conjugated onto the surface of silica coated CdSe@ZnS NPs via chemical conjugation. The LOD of this LFIA system was calculated to be 1.0754 ng/mL, which was lower than that of the gray zone for prostate cancer (4–10 ng/mL).

The characteristics of QDs are highly suitable for multiplexed detection, an important objective of LFIA. The importance of multiplexed detection is becoming increasingly emphasized in clinical diagnosis because there is often a need to detect one or more related biomarkers from identical samples to generate more conclusive information [50]. Wang et al. demonstrated a QD-based LFIA for the simultaneous quantitative detection of multiple tumor markers, alpha-fetoprotein (AFP), and carcinoembryonic antigen (CEA) [51]. The authors introduced two types of CdSe/ZnS core-shell QDs (546 nm and 620 nm) for the anti-AFP antibody and anti-CEA antibody, respectively. There was a single test line, which consisted of a mixture of mouse anti-AFP McAb, mouse anti-CEA McAb, and one control line. The LOD was 3 ng/mL and 2 ng/mL for two model biomarkers without evident cross-reactivity. The authors validated the tests using 130 clinical samples and the results exhibited high sensitivity (93% for AFP and 87% for CEA) and specificity (94% for AFP and 97% for CEA).

Wu et al. developed a QD-based LFIA to simultaneously detect two subtypes of influenza A (H5 and H9) [52]. The authors synthesized CdSe/ZnS core-shell QDs (620 nm) and functionalized them with influenza A virus subtype H5 antibodies and H9 antibodies, respectively. Two test lines for subtypes H5 and H9 were separately fabricated on the strip. The captured QD–antibody complex produced a bright fluorescent band in response to UV excitation at 365 nm. The authors acquired the images using three kinds of samples with various dilution factors, containing subtypes H5, H9, and a mixture of H5 and H9. The LOD of H5 and H9 subtypes was 0.016 HAU and 0.25 HAU, respectively. The specificity of the test was evaluated using other subtypes of type A influenza viruses (H1, H3, H5N1 re-4/6, H7N9, H9N2 re-2, and H9 SD696) and other viral antigens. They validated the assay with 47 clinical samples and reported a 100% match with a real-time PCR assay.

**Figure 4:**
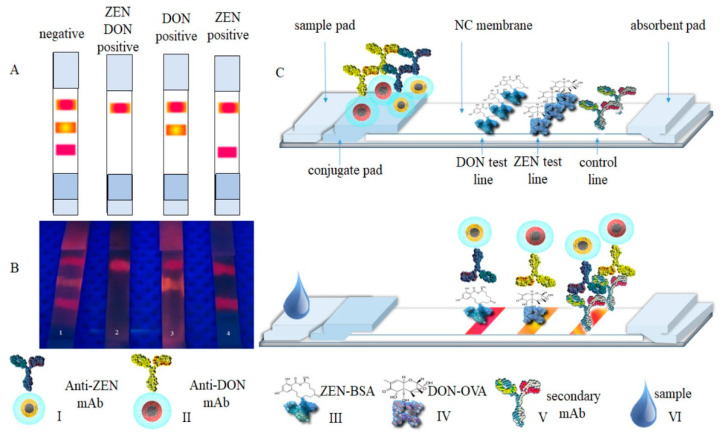
(**A**,**B**) Schematic and photograph of dual multicolor deoxynivalenol (DON) and zearalenone (ZEN) detection using LFIA. From left to right: (1) negative for ZEN and DON; (2) positive for ZEN and DON; (3) positive for ZEN and negative for DON; and (4) positive for DON and negative for ZEN. On the bottom: (I) CdSe_5.0nm_/CdS_6ML_-SiO_2_-anti-ZEN mAb conjugate, (II) CdSe_3.6nm_/CdS_6ML_-SiO_2_-anti-DON mAb conjugate, (III) ZEN-BSA conjugate, (IV) DON-OVA conjugate, (V) secondary anti-mouse mAb, and (VI) samples. (**C**) Schematic illustration of the LFIA test strip [53].

Goryacheva et al. fabricated two types of CdSe-based core-shell type QDs that emit orange and red colors, coating them with silica for application (Figure 4) [53]. An anti-deoxynivalenol antibody (Anti-DON) and an anti-zearalenone antibody (Anti-ZEN) were conjugated onto the surface of the QDs via chemical conjugation and developed with the samples. When the corresponding targets were present in the sample, the QDs were selectively immobilized onto each test line to generate the fluorescence signal. The cutoff values for zearalenone and deoxynivalenol in this LFIA system were 40 and 400 μg/kg, respectively.

Despite the excellence of QDs as a reporter, their toxicity often hinders their practical applicability. Wang et al. synthesized eco-friendly Cu:Zn−In−S/ZnS QDs for detecting tetanus antibodies [54]. The tetanus antigen and standard human IgG were modified on the test line and control line, respectively. The QD–goat anti-human IgG (Fc) was loaded on the conjugation pad. The LOD was 0.001 IU/mL, much lower than the minimum required level for protection (0.01 IU/mL) and the Au NP-based LFIA.

Shen et al. proposed an LFIA using QD beads which consisted of CdSe/ZnS QDs as amplification probes [55]. The QD bead size was estimated to be 247 ± 13 nm and they were conjugated with the anti-HBsAg monoclonal antibody. On the LFIA strip, the goat anti-HBsAg polyclonal antibody and donkey anti-mouse polyclonal antibody were coated on the test line and control line. The LOD of the assay was 75 pg/mL, which is much higher than that of the routinely-used Au NP-based LFIA. The authors also evaluated 96 clinical serum samples and compared the results with the commercial HBsAg ELSA kits. No false negative results were detected and the quantified results from the positive sample were comparable to the commercial HBsAg CLIA kits.

**Figure 5:**
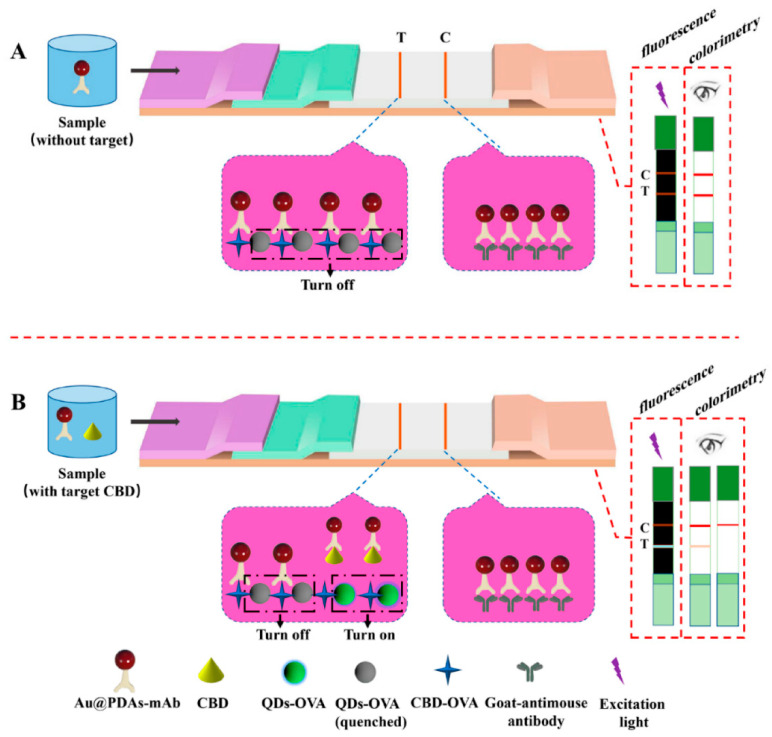
Principle of Au@PDAs–QDs–LFIA. (**A**) Detection mode without target carbendazim(CBD), and (**B**) detection mode with target CBD [56]. T and C indicate test line and control line, respectively.

Mao et al. reported an LFIA method in which QDs were not loaded onto the sample pad but were immobilized onto the test line (Figure 5) [56]. They immobilized QDs and carbendazim, as the target compound, onto the test line, coating with ovalbumin. Anti-carbendazim antibody-conjugated Au NPs (Au@PDA–mAb) were prepared and developed from a sample pad. In the absence of carbendazim in the sample, the carbendazim immobilized on the test line captured as much Au@PDAs–mAb as possible. Due to the captured Au@PDAs–mAb, the fluorescence signal of the test line was quenched and a deep red line was observed. In contrast, the number of captured Au@PDAs–mAb on the test line was reduced when carbendazim was present in the sample because Au@PDAs–mAb was already bound to carbendazim before development. Thus, the color of the test line was fainter and the fluorescence signal intensity increased, similarly to the number of unquenched QDs. The cutoff value for carbendazim was 0.0156 μg/mL in fluorescence analysis and 0.5 μg/mL in colorimetric analysis.

There has been continued attention to QD-based LFIA during the COVID-19 pandemic. Li et al. developed a QD-based LFIA for detecting SARS-CoV-2-specific antibodies [57]. For the probe preparation, the authors synthesized ZnCdSe/ZnS QDs and conjugated them with SARS-CoV-2 nucleocapsid (N) proteins. *Staphylococcus aureus* protein A (SPA) and mAb 4B8 antibody were coated on the test line and control line, respectively. The SPA is utilized as a capture molecule, targeting the Fc segment of SARS-CoV-2-specific antibodies. The LOD was 1:1.024 × 10^5^, with an IgG concentration of 48.84 ng/mL. No cross-reactivity was observed against the antibodies of other relevant coronaviruses and respiratory infection-related viruses. Zhou et al. also prepared highly luminescent QD nanobeads embodying CdSe/ZnS QDs into the polymeric matrix for detecting SARS-CoV-2 total antibodies [58]. To employ a double antigen assay, the authors conjugated QD nanobeads with SARS-CoV-2 spike proteins and separately prepared QD nanobeads labeled with mouse anti-digoxin antibodies. The test line and control line were coated with spike protein and mouse anti-digoxin antibodies, respectively. They confirmed the performance of the assay using 122 serum samples, including 69 positive and 53 negative samples validated with RT-PCR.

### 2.5. LFIAs with UCNPs

Organic dyes or QDs, both of which are widely used as fluorescence probes in bioimaging, emit distinct fluorescent light under irradiation with short-wavelength light, such as UV light. Although fluorescence imaging with UV light is useful, UV light can damage samples and induce autofluorescence owing to its absorption by biological samples. To overcome this problem, UCNPs, which can be excited by irradiation with long-wavelength light, have been developed [59]. Photo upconversion is the phenomenon that converts two or more low-energy excitation photons, generally near-infrared (NIR) light, into shorter wavelength emissions. The most interesting characteristic is the production of a higher energy anti-Stokes shift that allows us to separate photoluminescence (PL) from the excitation wavelength. The UCNPs comprise a crystalline host matrix and dispersed dopant as a guest ion. In this host–guest system, NaYF_4_ is the most preferred efficient host. Moreover, various dopant lanthanide ions (Nd^3+^, Ho^3+^, Er^3+^, Tm^3+^, and Tb^3+^) have been proposed as useful candidates for the guest and the emission depends on the choice of the activator and sensitizer ions. Most UCNPs absorb NIR light, which does not interfere with biological samples, and emit fluorescence. In addition, UCNPs have various optical properties similar to QDs, such as a narrow emission bandwidth, high photostability, long lifetime, and tunable emission [60,61].

Jin et al. optimized UCNP-antibody conjugates in terms of particle size and probe density through the systemic study [62]. For the optimization, the authors chose CRP as an example of LFIA. They first synthesized UCNPs (NaYF_4_:Yb^3+^ and Er^3+^) having various diameters (50 nm, 100 nm, 200 nm, and 500 nm) and conjugated with anti-CRP Ab#8 antibodies with various densities (from 90% to 10%). The LFIA strip was prepared with anti-CRP Ab7# antibody for the test line and goat anti-mouse IgG antibody for the control line. In this investigation, they found that LOD decreased with the particle size and increasing concentration of the probe. At an optimum condition, the LOD was 0.046 ng/mL, with a broad detection range between 0.2 and 300 ng/mL. Lastly, they validated the assay using 12 clinical samples and the detection results were consistent with the chemiluminescent immunoassay method.

Liu et al. reported a detection method for cephalexin (CEX) with luminescent UCNP-based LFIA [63]. They fabricated core-shell type NaGdF_4_:Yb,Er@NaGdF_4_ UCNPs via a seed-mediated method and conjugated an anti-cephalexin monoclonal antibody using click chemistry. The visual detection limit of CEX with the fabricated UCNPs was 10 ng/mL, similar to that of Au NPs. Detection of CEX with fabricated UCNPs showed linearity in the 0.5 to 100 ng/mL range and the LOD of CEX with UCNPs was determined to be 0.6 ng/mL. Given that this LOD was similar to that of Au NPs, the authors concluded that the fabricated core-shell-type UCNPs could be an alternative to Au NPs.

**Figure 6:**
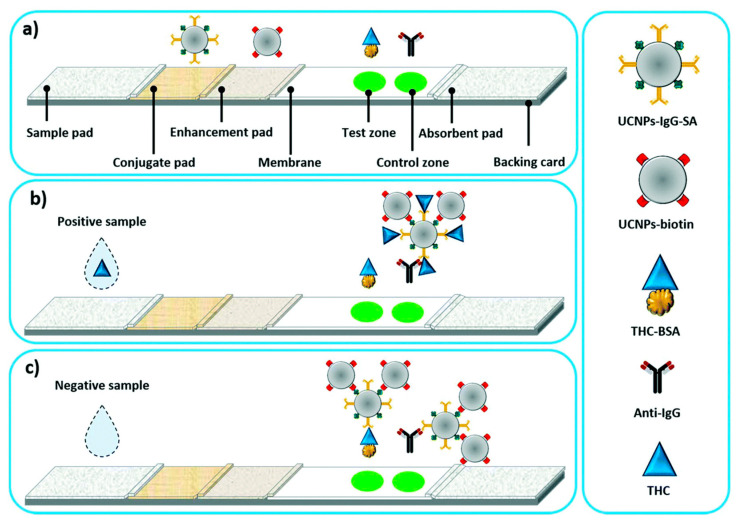
Upconversion of nanoparticle-based LFIA for the detection of tetrahydrocannabinol (THC). (**a**) Structure of the LFIA strip with the enhancement pad, (**b**) LFIA in the presence of THC, and (**c**) LFIA in the absence of THC [64].

Another application of UCNPs in LFIA analysis was reported by Chand et al. [64] (Figure 6). For the detection of tetrahydrocannabinol (THC), a biomarker for cannabis detection, IgG and streptavidin-conjugated UCNPs (UCNPs-IgG-SA) were prepared and loaded onto the conjugate pad of LFIA. Biotin-conjugated UCNPs (UCNP-biotin) were also prepared and loaded onto an enhancement pad next to the conjugate pad to enhance the fluorescence signal. In the presence of THC in the sample, UCNPs-IgG-SA were sequentially bound to THC and UCNP-biotin during development. As the IgG of the UCNPs complex was already bound to THC, the THC-bound UCNP complex was rarely immobilized on the test line coated with THC. Meanwhile, when THC was absent from the sample, the UCNPs complex could be immobilized on the test line and showed a fluorescence signal. This LFIA system for THC detection showed a 20% increase in intensity compared to the standard LFIA, with an LOD for THC at 2 ng/mL.

**Figure 7:**
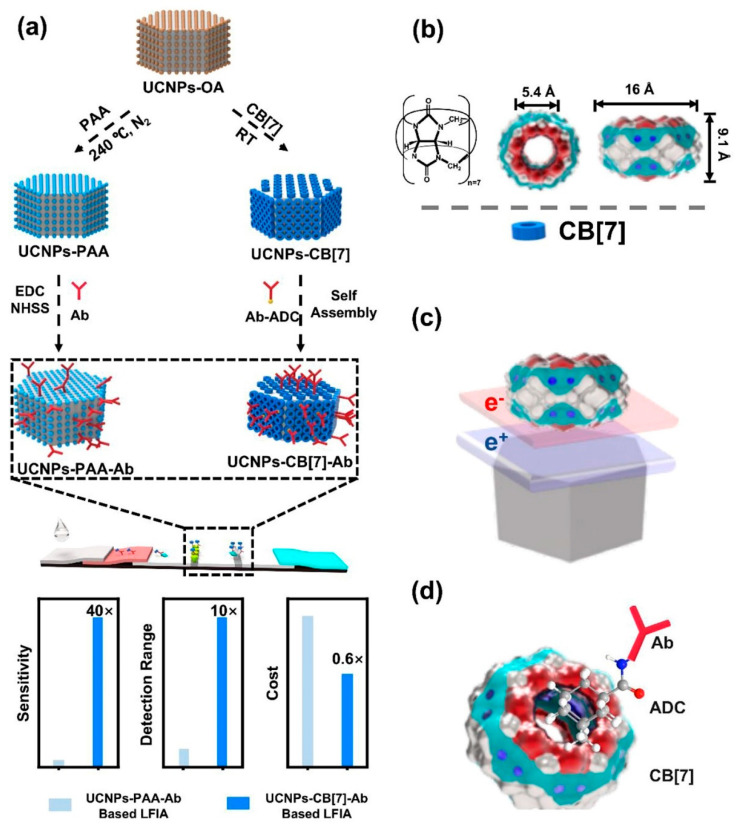
Schematic illustration of the upconversion nanoparticle (UCNP) probes used in an LFIA. (**a**) Preparation of the traditional and novel UCNP probes and the performance comparison of the two LFIAs. (**b**) Molecular structure and size of Cucurbit[7]uril (CB[7]). (**c**) CB[7] bound strongly with the NaYF_4_ crystal surface (Log K > 25.16) because of charge transfer interactions. (**d**) Conjugation of antibodies (Ab) via the host–guest supramolecular reaction between CB[7] and 1-adamantanecarboxyl (ADC) [65].

To improve the photoluminescence efficiency of UCNPs used in LFIA, Huang et al. modified the surface of UCNPs using a supramolecular self-assembly strategy (Figure 7) [65]. UCNPs were coated with cucurbit[7]uril (CB[7]) and bound antibodies for target recognition via host–guest interactions between CB[7] and 1-adamantanecarboxyl conjugated with antibodies. The prepared UCNPs showed high PL intensity, stability, and binding affinity for targets such as *Escherichia coli* O157:h7 or danofloxacin, even though less antibody was consumed during modification. CB[7]-coated UCNPs showed a 40-fold higher sensitivity and a 10-fold higher detection range in LFIA than UCNPs coated with polyacrylic acid.

Owing to the unique characteristics of UCNPs, the improvement of assay design has also been achieved. For example, Guo et al. developed an LFIA assay using orthogonal emissive UCNPs to suggest a solution for the double-line issue in LFIA [66]. The authors utilized lanthanide-ion-doped UCNPs, which possessed ladder-like energy levels, as signal reporters for both reporting signal and calibrating signals. They designed a dumbbell shaped UCNP, which displayed red and green emissions from the same activator Er^3+^ ion. Since the energy migration pathways could be manipulated under 980 nm and 808 nm excitations, the assay did not require a separate control line and acquired the results from the integrated test line. They utilized aflatoxin B1, a grain toxin, as a model target for rapid and quantitative detections. The LOD was 25 ng/mL with a linear range from 20 to 500 μg/mL. Moreover, they demonstrated an assay using a ring-assembled strip based on the single-line assay for improving detection capacity.

Even though a UCNP-based LFIA for detecting SARS-CoV-2 has not yet been reported, UCNPs are attractive probes for detecting infectious viruses. For example, Martiskainen et al. developed an assay for the detection of human immunodeficiency virus 1 (HIV-1) and 2 (HIV-2) antibodies [67]. Since anti-HIV antibodies indicate the body’s immune response to HIV infection, various commercial kits in the market detect those antibodies. The authors conjugated UCNPs with the HIV-1 and HIV-1 recombinant antigens. These antigens were coated on the test line, whereas HIV-1 gp41 rabbit polyclonal serum was coated on the control line. Martiskainen et al. also developed a UCNP-based LFIA for detecting HBsAg [68]. They conjugated UCNPs with mouse monoclonal anti-HBsAg antibody, which is in-house produced by hybridoma technology. The mixture of three types of anti-HBsAg antibodies, including two in-house and one commercial, was coated on the test line, and rabbit anti-mouse IgG was coated on the control line. The LOD of the assay was calculated to be 0.1 IU/mL in the spiked serum, comparable to the LOD of commercial LFIA kits. Finally, they compared their UCNP-based LFIA with commercial chemiluminescent immunoassay.

### 2.6. LFIAs with Silica Template-Based NPs

**Figure 8:**
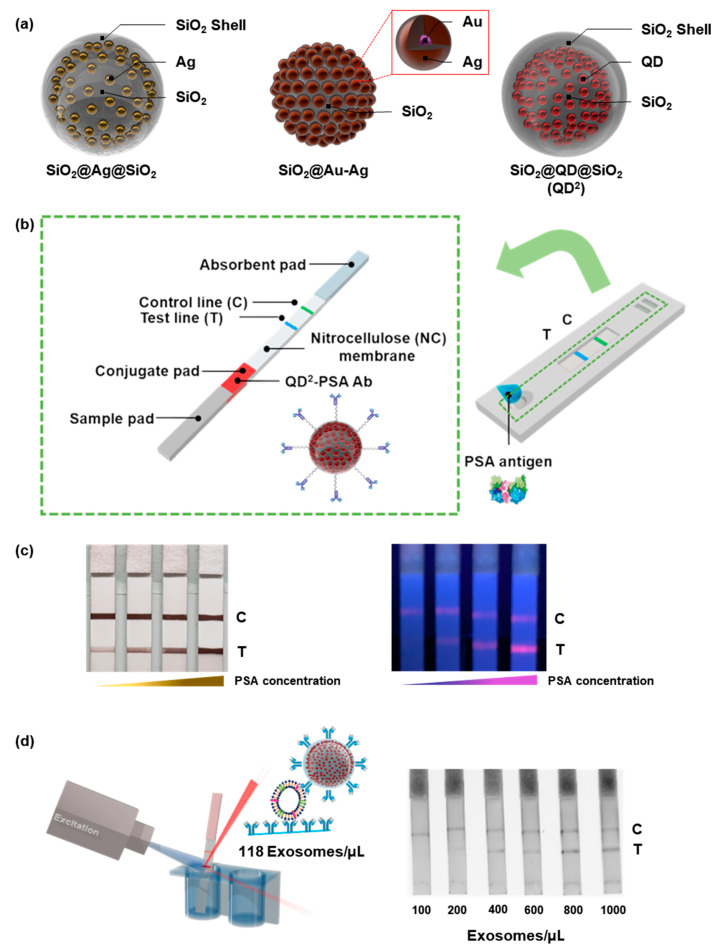
The LFIA systems using silica template-based NPs (**a**) Illustration of silica NPs [69,70,71]. (**b**) Schematic illustration of LFIA system for PSA detection with silica NPs [71]. (**c**) Detection of PSA via LFIA with SiO_2_@Au-Ag (**left**) and QD^2^ (**right**) [70,71]. (**d**) Schematic illustration of an LFIA system for exosome detection with QD^2^ (**left**) and results from exosome detection with the LFIA system (**right**) [72]. T and C indicate test line and control line, respectively.

Despite their utility, NPs of only a few nanometers in size are difficult to handle during surface modification. In addition, enhancement of the signal intensity is necessary for sensitive detection because of the weak signal intensity of individual NPs. To overcome these problems, our group developed a system in which metal NPs, such as silver, QDs, or Au/silver alloys, were embedded in a silica NP template (Figure 8) [69,70,71]. The signal intensity of fabricated individual fabricated NPs was markedly enhanced because of the numerous embedded metal particles compared with that of single metal NPs.

Kim et al. prepared silica-encapsulated silver-assembled silica NPs (SiO_2_@Ag@SiO_2_ NPs) and detected PSA [69]. Thiol-modified silica NPs, of approximately 150 nm in size, were used as templates. Finally, SiO_2_@Ag@SiO_2_ NPs were prepared by covering the fabricated NPs with a silica shell to prevent contact with the silver NPs. During the formation of silver NPs, the concentration of the silver precursor was varied to control the amount and size of the embedded silver NPs. For SiO_2_@Ag@SiO_2_ NP_2.6_, the highest concentration of the silver precursor exhibited a light gray color when dispersed in ethanol and a light pink or yellow color when the concentration of the silver precursor was low. Based on their enhanced LSPR effect, which originated from the narrow gap between the embedded silver NPs, SiO_2_@Ag@SiO_2_ NP_2.6_ showed a strong optical signal intensity when dropped onto the nitrocellulose membrane. With SiO_2_@Ag@SiO_2_ NP_2.6_, PSA could be detected within 10 min with an LOD of 1.1 ng/mL.

Au-silver-assembled NPs have also been embedded in silica templates to fabricate optical nanoprobes [70]. Pre-synthesized Au NPs (7 nm in size) were attached to the surface of silica NPs and silver NPs were added around the attached Au NPs. These NPs (SiO_2_@Ag–Au NPs) exhibited a deep gray color when dispersed in ethanol. They had stronger optical intensity than Au NPs when the same concentration of particles was dropped onto the nitrocellulose membrane. SiO_2_@Au–Ag NPs enabled the visual detection of PSA in clinical samples via LFIA and a semi-quantitative analysis was also possible.

QD-embedded silica-encapsulated NPs (SiO_2_@QD@SiO_2_ and QD^2^) were also used as optical nanoprobes in LFIA [71,72]. QDs^2^ were prepared by attaching QDs onto thiolated silica NPs and silica encapsulation [73]. QD^2^ showed an approximately 200-fold stronger PL intensity than single QDs because many QDs were embedded in silica templates. Bock et al. conjugated anti-PSA antibodies onto the surface of QDs^2^ and used them to detect PSA via LFIA [71]. After the development of samples, a photograph of LFIA strips was captured under a 365 nm UV lamp and the signal intensities were evaluated using the ImageJ program. The LOD of LFIA for PSA was calculated as 0.138 ng/mL, far lower than the “safe zone” threshold for prostate cancer (2.5 ng/mL) [74]. In addition to good sensitivity, the fabricated LFIA kit showed high selectivity such that the kit did not exhibit a signal at the test line with alpha-fetoprotein or newborn calf serum. With this superior performance, 47 clinical samples were analyzed using the LFIA kit. The resulting area under the curve was 0.852, with no false-negative results. Kim et al. applied QDs^2^ to LFIA for detecting exosomes [72]. Anti-CD63 antibodies, which can bind to exosomal CD63, were immobilized onto the surface of QDs^2^ to capture exosomes in the sample. The calculated LOD was 118 exosomes/μL, approximately 11 times lower than the previous results for detecting PSA with LFIA using Au NPs [75], double Au NP conjugates [76], and Au-palladium NPs [77].

Other research groups also reported silica template-based methods for fabricating LFIA probes. Lu et al. fabricated spherical core-shell gold-silica nanoparticles (AuNP@SiO_2_ NPs) by silylation of surfactant-stabilized Au NPs [78]. The authors utilized AFP as a model biomarker. Based on the highly stable NP, the LOD of the assay was down to 300 pg/mL, comparable to that of ELISA.

During the COVID-19 pandemic, the LFIA development using a silica template-based method was also achieved. Wang et al. developed colorimetric-fluorescent dual-mode LFIA for detecting IgM and IgG in human serum [79]. The authors decorated both Au NPs and CdSe/ZnS QDs on the SiO_2_ NPs to fabricate SiO_2_@Au@QD nanobeads with the help of polyethylenimine layers. Thereafter, SARS-CoV-2 spike proteins were conjugated on the surface of the SiO_2_@Au@QD nanobeads. The anti-human IgG and anti-human IgM antibodies were separately coated on two test lines, whereas the anti-S antibody was coated on the control line. In the fluorescence signal, the assay was 100 times more sensitive than commonly used Au-based LFIA for SARV-CoV-2-specific IgM/IgG detection. The colorimetric-fluorescent dual-mode detection was helpful in the rapid (colorimetric) and more accurate and quantitative detection (fluorescent). The authors validated 57 clinical samples (16 positives and 41 negatives) and identified 100% of patients with 100% specificity. Since the IgG level, IgM level, and IgG/IgM ratio are valuable indicators of the patient’s infection history, the simultaneous detection of IgG and IgM can not only contribute to the accurate identification of a SARS-CoV-2-infected person but can also monitor the progress of the disease.

## 3. Conclusions and Future Directions

The LFIA is considered the most promising diagnostic tool because it is simple, rapid, and low-cost. Compared with other diagnostic methods, such as ELISA, LFIA can be used without the need for expensive or uncommon instruments. Furthermore, quantitative/qualitative analysis of target biomarkers is enabled by the use of photographic images. However, low sensitivity and reproducibility often limit its application. The recent advances based on the engineering of optical NPs lay the foundations for more sensitive and accurate LFIA.

In this review article, we have discussed LFIA systems with various types of optical NPs, including Au NPs, carbon NPs, QDs, UCNPs, and silica template-based NPs. These systems have successively been developed and have shown potential for detecting clinically relevant targets, such as cancer biomarkers, including AFP, CEA, and PSA. Researchers have highlighted the potential of LFIA for biomarker detection at an early disease stage, even when a little amount of sample is analyzed. Enhanced LFIA is to be of particularly value in diseases where early detection is essential essential [80], such as pancreatic cancer. Therefore, we expect rapid, sensitive, convenient, and accurate diagnoses of diseases when optical NPs are optimized for a given target.

In addition, the present COVID-19 pandemic highlights the important role of LFIA. In this urgent situation evolved by a highly contagious virus, the short sample-to-result time and user-friendly procedure of LFIA enabled us to conduct mass-scale self-testing. Although the sensitivity of these commercial kits was somewhat unsatisfactory, they have contributed to preventing the spread of SARS-CoV-2. The health threat of disease, from various recognized diseases to emerging unknown diseases such as COVID-19, continues. There is expected to be an increasing demand for rapid and convenient diagnostics in the future. There are several requirements for the future role of FIA-based diagnostics: (a) as this review article described, the advancement in probes will be required for sensitive, stable, and reproducible detection and (b) the accurate quantification in the range of disease-related marker concentration is also crucial. The first step to achieving both requirements is the development of improved probes. Therefore, it is essential that research on optical NPs used in LFIA is continued.
